# The Visual Priming of Motion-Defined 3D Objects

**DOI:** 10.1371/journal.pone.0144730

**Published:** 2015-12-14

**Authors:** Xiong Jiang, Yang Jiang, Raja Parasuraman

**Affiliations:** 1 Department of Neuroscience, Georgetown University Medical Center, Washington, DC, 20007, United States of America; 2 Department of Behavioral Science, University of Kentucky College of Medicine, Lexington, KY, 40506, United States of America; 3 Department of Psychology, George Mason University, Fairfax, VA, 22030, United States of America; Vrije Universiteit Brussel, BELGIUM

## Abstract

The perception of a stimulus can be influenced by previous perceptual experience, a phenomenon known as perceptual priming. However, there has been limited investigation on perceptual priming of shape perception of three-dimensional object structures defined by moving dots. Here we examined the perceptual priming of a 3D object shape defined purely by motion-in-depth cues (i.e., Shape-From-Motion, SFM) using a classic prime-target paradigm. The results from the first two experiments revealed a significant increase in accuracy when a “cloudy” SFM stimulus (whose object structure was difficult to recognize due to the presence of strong noise) was preceded by an unambiguous SFM that clearly defined the same transparent 3D shape. In contrast, results from Experiment 3 revealed no change in accuracy when a “cloudy” SFM stimulus was preceded by a static shape or a semantic word that defined the same object shape. Instead, there was a significant decrease in accuracy when preceded by a static shape or a semantic word that defined a different object shape. These results suggested that the perception of a noisy SFM stimulus can be facilitated by a preceding unambiguous SFM stimulus—but not a static image or a semantic stimulus—that defined the same shape. The potential neural and computational mechanisms underlying the difference in priming are discussed.

## Introduction

Perceptual priming refers to the facilitation or biasing of the perception of a stimulus by previous experience with a similar stimulus. For example, in the case of object *repetition priming* (for reviews, see [1–4],) a previously encountered stimulus is recognized faster and more accurate than a novel stimulus. Such priming effects (or perceptual memory) have been attributed to modulations of neuronal responses at corresponding brain regions, as revealed in both brain imaging [5–9] and transcranial magnetic stimulation (TMS) studies [10–12].

In contrast to the static visual stimuli used in most visual perceptual priming studies (e.g., [13]), our visual world contains many three-dimensional (3-D) complex objects that are constantly moving, often in the presence of strong environmental noise, making object recognition sometimes ambiguous and challenging. Visual motion provides important information for perceiving object structures and surfaces in the visual world. For instance, 3D perception can be recovered from 2D images, a phenomenon known as the kinetic depth effect (KDE), or structure from motion (SFM) [14]. When an SFM stimulus is presented in the absence of depth cue, its rotating direction is ambiguous and can be perceived to be rotating in either direction. Furthermore, its perceived rotating direction correlates with neural activations in the MT/V5 [15] and other dorsal brain regions [16], and can be biased by a preceding stimulus [17–20]. This motion priming (or motion inertia) phenomenon has been studied extensively. For instance, while previous studies found that the change of object shapes have little to no effect on 3-D motion priming [21,22]–suggesting a largely disjointed neural representation of motion and object structure of SFM stimuli; recently we and others have found reduced motion priming when the object shapes are different between the prime and target SFM stimuli [18,20]–suggesting a more integrated neural representation of motion and object structure of SFM stimuli. In addition, using ambiguous SFM stimuli, another recent functional magnetic resonance imaging (fMRI) study revealed separate neural mechanisms underlying stimulus repetition and perceptual repetition [9]. However, little is known about whether and how the object recognition of an SFM stimulus can be primed by a preceding stimulus.

To address this question in the present study, we investigated whether the object recognition of an SFM stimulus can be biased/facilitated by a preceding visual stimulus using a classic prime-target paradigm: the target SFM stimuli were always transparent rotating 3-D objects that were difficult to recognize due to a high percentage of noise dots added to the stimulus presentation, and the prime stimuli were either a set of random dots, an SFM stimulus with unambiguous shape and motion, a static object shape, or a semantic word that defines an object shape. In addition, using varying prime-target interval, we examined the time course of object priming of SFM stimulus when it was preceded by another unambiguous SFM stimulus.

## General Methods

### Participants

Participants were undergraduate and graduate students (age 18–30) recruited from local institutes, who were not aware of the purpose of study before finishing the experiments and only participated in one of the three experiments. All had normal or corrected to normal (20/20) visual acuity. There were fourteen participants in Experiment 1, eleven in Experiment 2A, and nine in Experiment 2B, ten in Experiment 3A, and nine in Experiment 3B. Data from two participants were excluded due to chance-level performance in all conditions. Experimental procedures were approved by the Catholic University of America Institutional Review Board, and written informed consent was obtained from all participants prior to experiment.

### Visual Stimuli

Each stimulus consisted of a set of dots inside of an invisible 16cm x 16cm square. In all conditions, a prime stimulus was followed by the target stimulus, with an intervening inter stimulus interval (ISI) (130 and 800 milliseconds in Experiment 1, 500, 2000, 4000, and 6000 milliseconds in Experiment 2, and 2000 milliseconds in Experiment 3). The stimuli were chosen so that when presented alone, the perception of the first (prime) stimuli was unambiguous (100% accuracy with no noise dots), whereas perception of the second (target) stimuli was challenging and uncertain (always less than 100% accuracy due to the presence of a high percentage of noise dots).

Two types of (non-object related) prime stimuli were used in the control conditions (as defined in the *Procedure and Design* section). Each stimulus was depicted by 200 luminance dots that were randomly picked within the square space (16cm x 16cm). Half of the dots were high in luminance, and the other half were low in luminance. The luminance levels were set through OpenGL, with the high luminance at the value of 255 (the highest possible value, and 0 was the lowest possible value), and the low luminance at the value of 80. In the control condition using static dots as the prime, the set of dots remained in the same position on the screen for 550 milliseconds. In the random motion control condition, the set of dots moved randomly in 2-D space without generating any explicit object perception; such random apparent motion was depicted by 10 animation graphic frames, and each frame was displayed for about 50 milliseconds.

**Fig 1:**
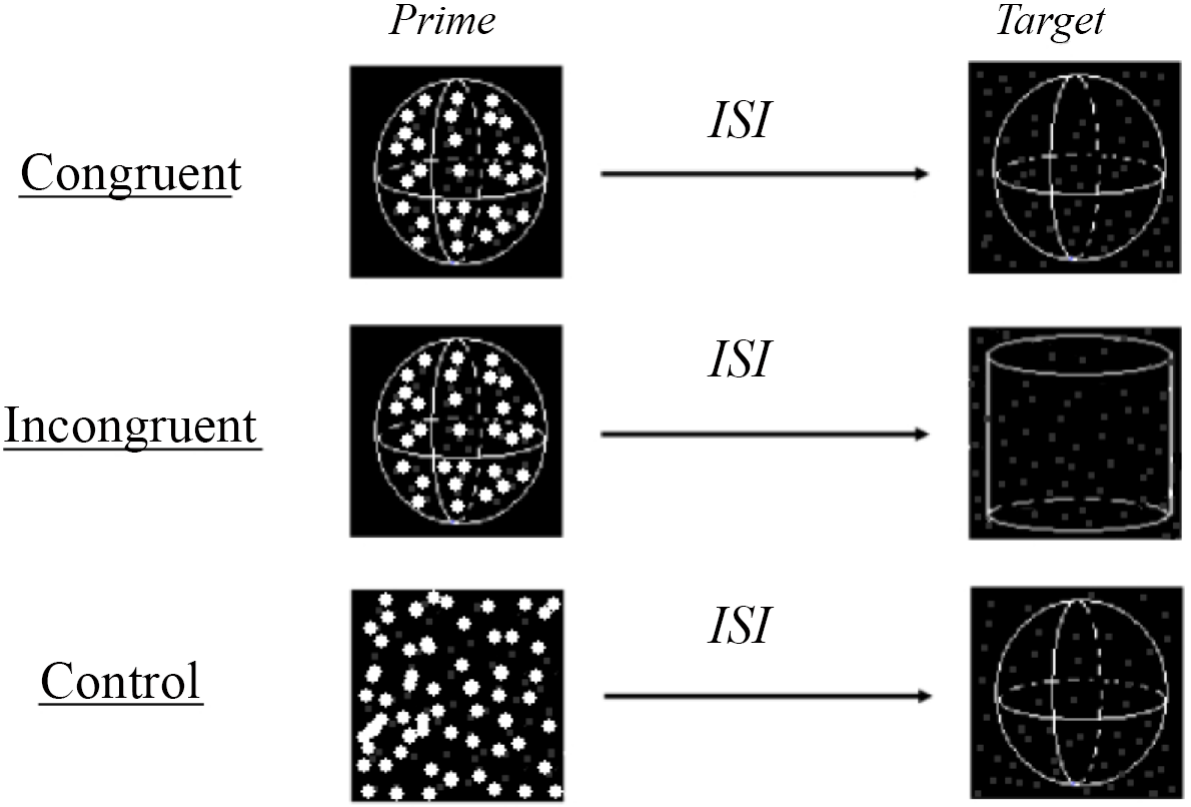
The scheme of paradigm and stimuli used in the present study. Two Structure-From-Motion stimuli were used in the present study, sphere and cylinder. The SFM stimuli displayed as the prime stimuli were unambiguous and only with dots from the projection of the surfaces (cylinder or sphere), while the SFM stimuli displayed as the target stimuli were ambiguous and with a high percentage of noise dots. There are three priming conditions used in the present study, congruent (when the prime and target SFM stimuli defined same object), incongruent (when the prime and target SFM defined different object), and control (when the prime stimuli were random dots). For prime stimuli, the dots in the front were larger (3:1) and brighter (4:1) than the dots in the back. For the target stimuli, all dots had the same size and luminance as the dots in the back of prime stimuli. It might be worth to note that during the experiment, there was no drawing of objects as shown here (for illustration purpose).

There were two types of SFM primes that were used in the congruent and incongruent conditions (as defined in the *Procedure and Design* section). Two hundred dots were randomly chosen from the surface of either a sphere (radius = 7.4cm), or a cylinder (radius = 7.2cm, height = 7.8cm) (Fig 1). The dots rotated around the Y-axis, so that participants perceived an unambiguous and vivid 3-D object (either a sphere or a cylinder) when it was rotating. The direction of rotation was either clockwise or counterclockwise. Each frame remained on the screen for about 50 milliseconds. Each set of dots on two successive frames differed 5° from each other in location (i.e., rotating 5° around Y-axis in clockwise or counterclockwise direction). In total, 10 animation frames were displayed per trial. Dot luminance changed according to position with two luminance levels (high luminance for front dots, low luminance for back dots). The number of high and low luminance dots was roughly equal across all conditions.

There were two types of SFM stimuli were used as the target stimuli (Fig 1). Each target stimulus included two sets of dots. One set of dots was randomly chosen from the surface of a rotating sphere or cylinder that was similar to those used as prime but slightly differed from the prime in dimension (range -5% to +5%, to avoid low level adaptation); the other set consisted of noise dots that were chosen inside the square and were used to eliminate the boundaries of the objects (sphere or cylinder). The dots from the object (sphere or cylinder) rotated at the same speed as the unambiguous prime stimuli. The total number of dots (signal plus noise dots) was kept consistently at 300, and the ratio of signal dots to noise dots was about 0.265. The size of both high and low luminance dots was one pixel, which was about 0.03° when viewed from a distance 57 centimeters. All the dots had the same low luminance as those used in the prime stimulus. The signal dots in the prime stimuli had a lifetime of ten frames (the entire stimuli presentation time, see above). In contrast, by average, the signal dots in the target stimuli had a lifetime of about 7.6 frames.

### Procedure and Design

For each trial, there were two stimuli presented successively with an ISI—the prime and the target. Subjects were instructed to passively view the prime stimulus, and to identify the shape of (sphere versus cylinder) of the target stimulus by pressing the left- or right-arrow key that corresponded to each type of object. All subjects were asked to respond on every trial even if they did not perceive an object clearly.

Fig 1 illustrates the three experimental conditions—the *control condition*, in which the prime stimuli were either static or moving random dots (both static and moving random dots were used in Experiment 1, while only static random dots were used in Experiments 2A & 2B); the *congruent condition*, in which the prime and target stimuli were the same objects (sphere-sphere or cylinder-cylinder); and the *incongruent condition*, in which the prime and target stimuli were different objects (sphere-cylinder or cylinder-sphere). The delay (ISI) between the prime and the target stimuli differed across the three experiments. To avoid inter-trial interference, the delay between trials was held constant at eight seconds in all experiments.

All the experiments were run on a Gateway PII computer with a Gateway GV700 monitor. The luminance and contrast of the monitor were both set to the highest values, and the refresh rate of the monitor was set to 60Hz. All stimuli were presented using customized code written in C with OpenGL, and were viewed from a distance of 57 centimeters.

### Data Analysis

Repeated measures ANOVAs and t-tests were used to analyze the behavioral data from the three experiments. Because the task was difficult (overall accuracy was lower than 80%), no reaction time (RT) demands were placed on participants, and there was no significant difference in RT between the congruent and incongruent condition, except in Exp. 2A with the ISI of 2s (p = 0.041, uncorrected).

## Experiment 1

This experiment investigated whether the perception of motion-defined 3D shapes could be primed or biased by previous perception of another motion-defined object. The shapes of the target SFM stimuli were defined exclusively by motion cues.

### Method

Two ISIs (130 and 800 milliseconds) were introduced between the prime and the target under the three conditions (*control*, *congruent*, *and incongruent*). Each participant performed 144 trials in total (48 trials for each condition). The order of ISIs was blocked and counter-balanced across all subjects. There were two control conditions: static dots or randomly moving dots.

### Results and Discussion

**Fig 2:**
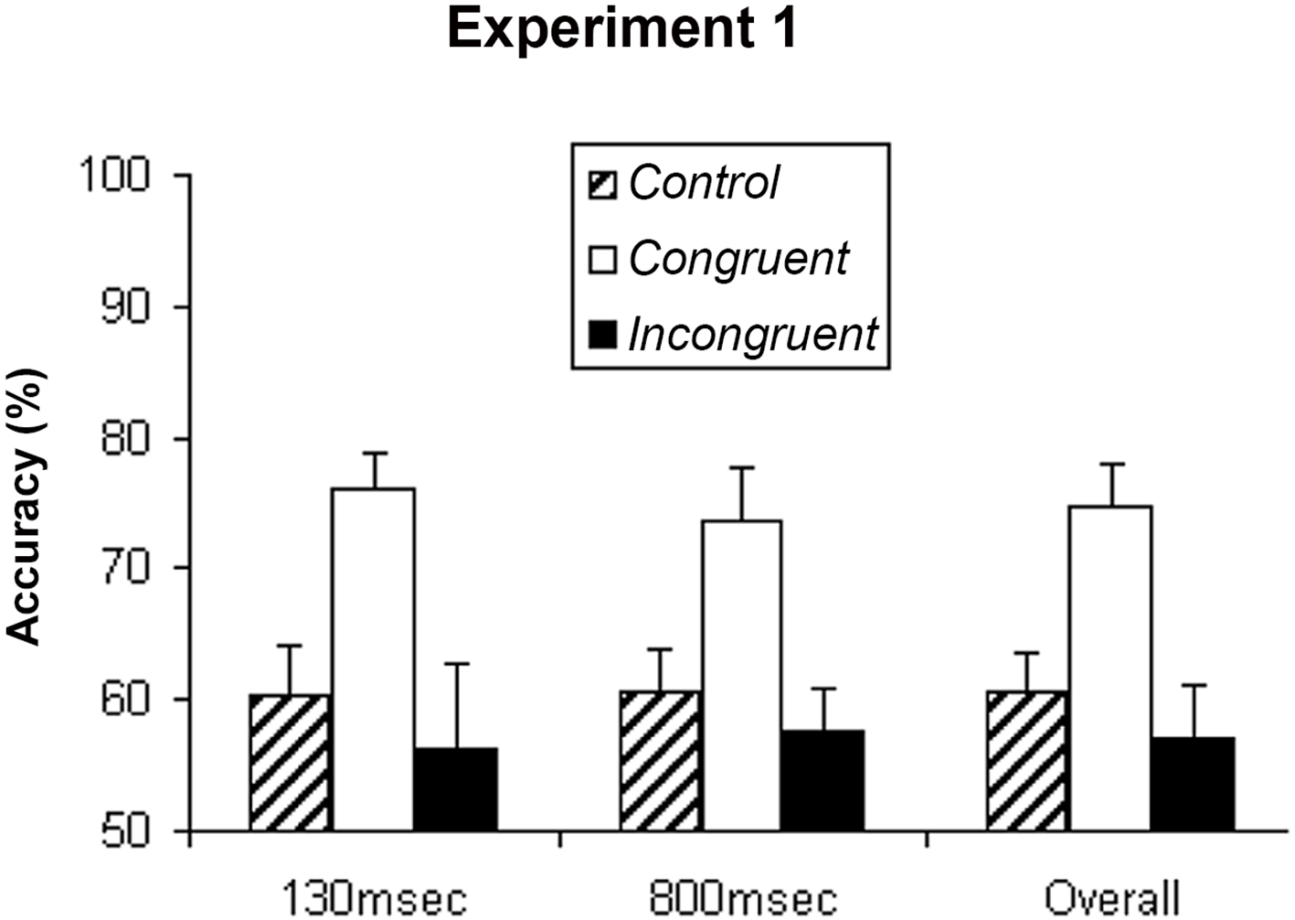
Experiment 1. Mean accuracy of the shape judgment about the target SFM stimuli from fourteen participants in Experiment 1. The data showed that participants could perceive the object of the target Structure-From-Motion (SFM) stimuli more accurately under congruent condition than the other two conditions (control and incongruent), while ISI did not affect their performance significantly. Thus, the shape perception of SFM stimulus can be primed by previous exposure to a SFM that defined the same shape. Error bars show standard error of mean (SEM).

Overall, when the target stimuli were preceded by static or random moving dots *(control condition)*, participants reported the shape of the target stimuli correctly on 60.6% of trials (SEM = 3.1%). If there is no priming effect on shape perception of SFM stimulus, then seeing the prime or not should not change the percentage of shape judgment of the target stimuli. However, we found that when the target stimuli were preceded by the unambiguous SFM stimuli that had the same shapes *(congruent condition)*, participants reported the shape correctly on 74.8% of trials (SEM = 3.1%). When the target stimuli were preceded by the SFM stimuli that had different shapes (incongruent condition), participants reported only 57% (SEM = 4.1%) of the shapes correctly (see Fig 2).

Because there was no significant difference between the two control conditions, the data from them were combined in further analysis. Repeated measures analysis of variance (ANOVAs) were carried out with the following factors: priming conditions (control vs. congruent vs. incongruent), and ISIs (130msec vs. 800msec). Significant effects were found among three conditions, F(2,26) = 8.925, p = 0.001, but not between the two ISIs, F(1,13) = 0.007, p = 0.937, and not the interaction between the priming conditions and ISIs, F(2,26) = 0.171, p = 0.843. Post hoc analysis revealed no significant difference between the control and incongruent conditions, F(1,13) = 1.073, p = 0.319, but significant differences between the congruent and each of the other two conditions (p<0.01 for both comparisons). In other words, when the prime and target SFM stimuli had the same structure (i.e., sphere-sphere, or cylinder-cylinder), object perception of the target SFM stimuli was facilitated, but when they had different structures (i.e., sphere-cylinder, or cylinder-sphere), accuracy did not differ from the control condition (p>0.53).

The results of Experiment 1 revealed that shape perception of a “cloudy” SFM stimulus (due to the presence of a high percentage of noise dots) was facilitated (primed) by prior exposure to an unambiguous SFM stimulus of a similar shape (Fig 1), but not an unambiguous SFM stimulus of a different shape, which did not differ from the neutral stimuli.

## Experiment 2

Experiment 1 found a perceptual priming effect of motion-defined objects. Like most priming phenomena, this effect could be time sensitive. The next logical issue therefore was to examine the time course of the priming effect. In the Experiment 2, we systematically varied the time between the prime-target interval (500–6000 ms) to explore the time course of the perceptual priming effect of SFM.

### Method

In Experiment 2, longer ISIs were used for both congruent and incongruent conditions to examine how long the priming effect would last. In particular, two ISIs (2000 and 4000 ms) were used in Experiment 2A, and four ISIs (500, 2000, 4000, and 6000 ms) were used in Experiment 2B. Only static random dots were used as the control condition in Experiment 2A & 2B. Experiment 2A, the control condition was conducted under a fixed ISI (two seconds), while in Experiment 2B, the control condition was conducted under all four ISIs. For each condition and ISI, 48 trials were administered to each participant in Experiment 2A, and 16 trials in Experiment 2B.

### Results and Discussion

#### Experiment 2A

**Fig 3:**
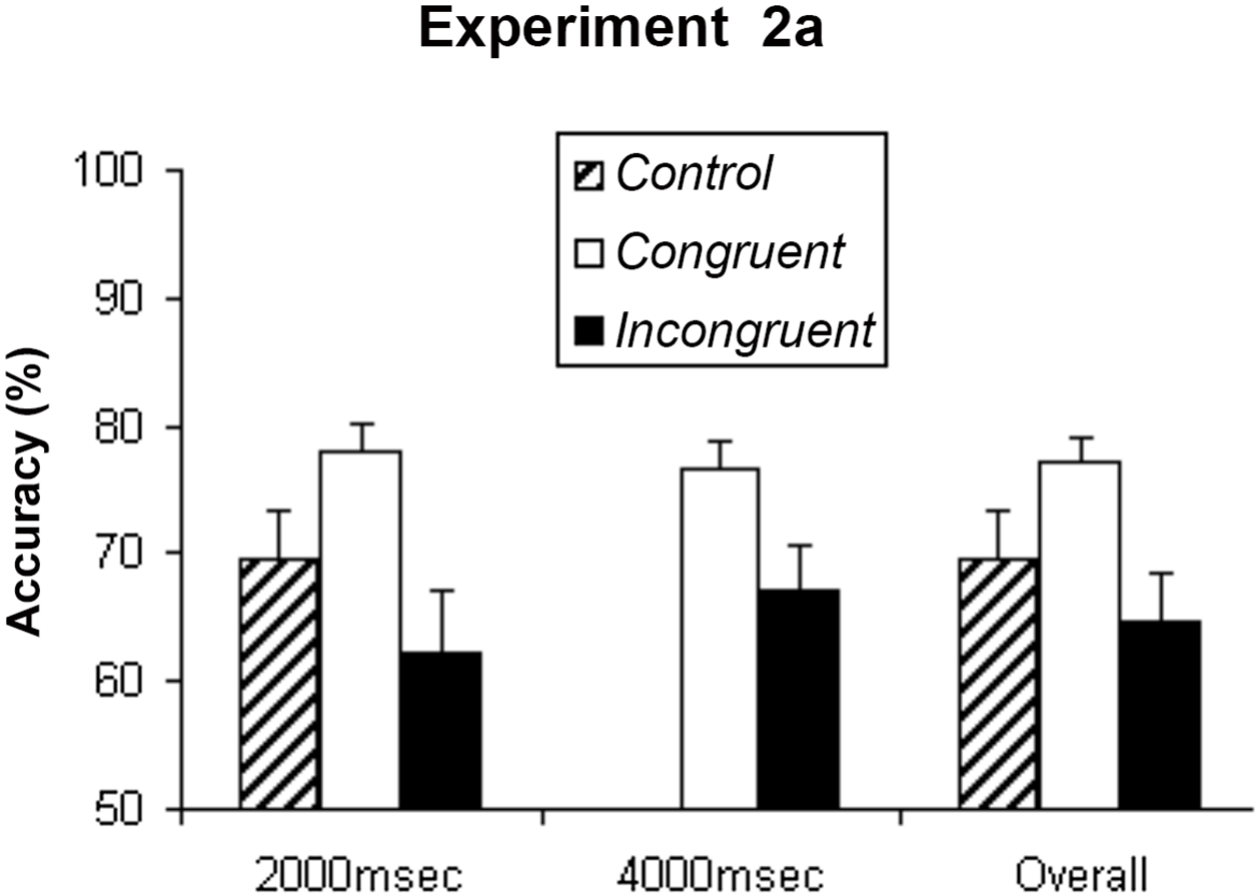
Experiment 2A. Mean accuracy of the shape judgment about the target SFM stimuli from eleven participants in the Experiment 2A. The data for the control condition was only recorded when ISI was two thousand milliseconds. Again, perceptual priming was found as indicated by the highest accuracy under congruent condition, and such priming effect became smaller with longer ISI (ISI = 4000 milliseconds), but still significant. Error bars show standard error of mean (SEM).

Overall, participants reported 69.7% (SEM = 3.8%) of target shapes correctly under the control condition (ISI = 2000 ms only). In contrast, under the congruent condition, participants reported 78% (SEM = 2.2%) (ISI = 2000 ms) and 76.5% (SEM = 2.2%) (ISI = 4000 ms) of target shapes correctly, and under the incongruent condition, participants reported 62.1% (SEM = 4.9%, ISI = 2000 ms) and 67.2% (SEM = 3.4%, ISI = 4000 ms) of target shapes correctly (see Fig 3).

Repeated measures ANOVA were carried out with the following factors: priming conditions (congruent vs. incongruent), and ISIs (2000msec vs. 4000msec). The analysis indicated that participants performed more accurately under the congruent condition than under the incongruent condition, F(1,10) = 9.862, p = 0.011. The difference between the congruent and incongruent conditions was larger when the ISI was shorter (2 seconds) than when it was longer (4 seconds), F(1, 10) = 5.360, p = 0.043, but the effect of ISI alone was not significant, F(1,10) = 0.717, p = 0.417. Post hoc tests revealed that the priming effect was significant at the longer ISI (p<0.05). In other words, when the prime and target SFM stimuli had the same structure, the object perception of the target SFM stimuli was facilitated even with an ISI up to four seconds, but the priming effect was diminished somewhat compared to the two-second ISI condition. Because the control condition was run with a fixed ISI of two seconds, it could not be included in the overall ANOVA. However, post hoc analysis indicated that, similar to Experiment 1, there was no significant difference between the control and incongruent condition when the ISI was two seconds (P>0.05).

#### Experiment 2B

**Fig 4:**
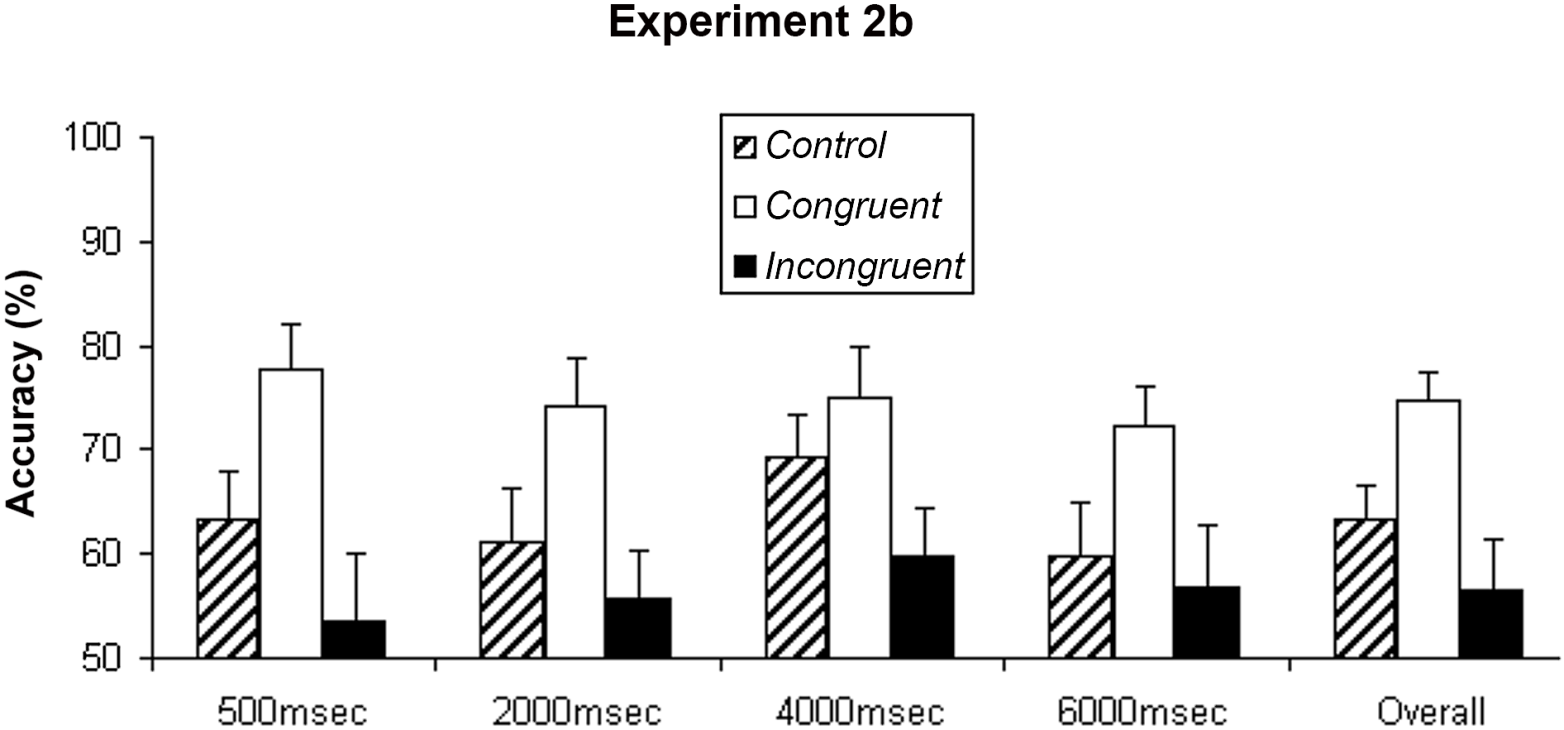
Experiment 2B. Mean accuracy of the shape judgment about the target SFM stimuli from nine participants in the Experiment 2B. This data not only found the priming effect again, but also show such priming effect started to decay after two seconds, and such decay was small and smooth. Error bars show standard error of mean (SEM).

In Experiment 2B, we tested the time course of the priming effect with a wider range of ISIs, from 500 milliseconds to 6 seconds. Overall, participants reported 63.4% (SEM = 3.3%) of target shapes correctly under the control condition. In contrast, under the congruent condition, participants reported 74.8% (SEM = 2.6%) of the target shapes correctly, and under the incongruent condition, 56.4% (SEM = 4.9%). Performance under the different ISIs is shown in Fig 4.

Repeated measures ANOVA were carried out with the following factors: priming conditions (control, congruent, and incongruent), and ISIs (500, 2000, 4000, 6000msec). The analysis indicated the main effect of the priming conditions was significant, F(2,20) = 13.205, p<0.001, but the effect of ISI was not significant, F(3,30) = 1.847, p = 0.160, nor was the interaction between ISI and priming condition, F(6,60) = 0.863, p = 0.527. Post hoc tests revealed that the priming effect was significant with the three short ISIs (p<0.01 for 500 and 2000 milliseconds, and p<0.05 for 4000 milliseconds), and approached a significant level with the longest ISI (6000 milliseconds) (p = 0.066). But when comparing congruent and incongruent conditions only, the priming effect was still significant even when the ISI was as long as 6000 milliseconds (p<0.05). That is, when the prime and target SFM stimuli had the identical structure, object perception of the target SFM stimulus was facilitated even with an ISI up to six seconds. The priming effect might have showed some degree of tendency to decline as the ISI increased, but this trend was not fully reliable within the range of ISIs tested.

Because overall performance levels were somewhat low in some of the priming conditions, we did a t-test for each condition in all experiments, but the data from different ISIs were collapsed into a single condition. Except for performance under the incongruent condition in Experiment 1 and 2B (p = 0.116 and 0.229 separately), accuracy performance under the other conditions in all experiments was significantly higher than chance level (50%) (at least p<0.01, and for most of them p<0.001).

**Fig 5:**
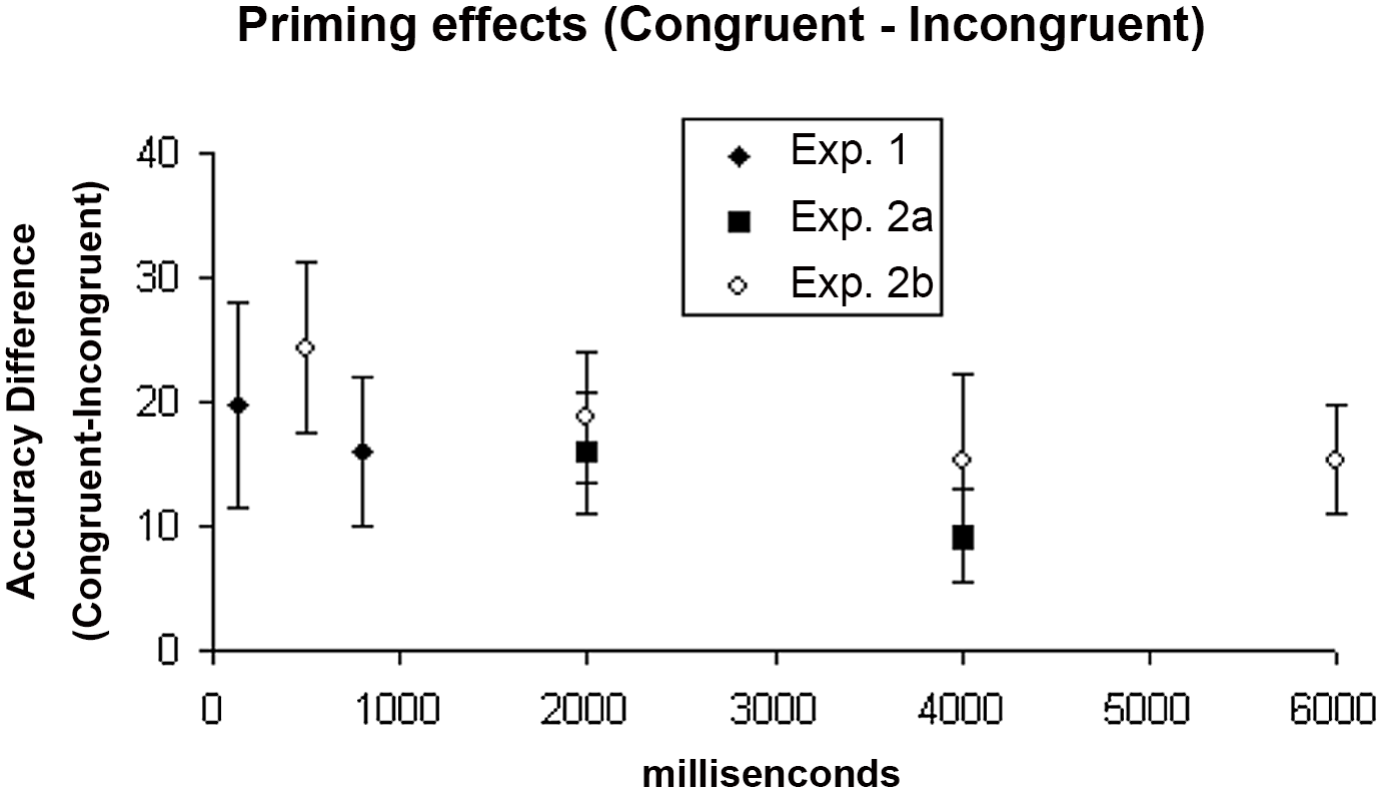
The priming effects of Exp. 1, 2A, and 2B. The priming effect indicated as the accuracy difference between the congruent and incongruent conditions across Experiment 1, 2A, and 2B, instead of difference between congruent and control conditions due to the lack of control conditions under several ISIs. From the figure, it was clear there was a such tendency that priming effect decayed with as the ISI increased, but such decay was small and smooth. The priming effect was smaller but more stable after a few seconds. Error bars show standard error of mean (SEM).

Furthermore, we calculated the accuracy difference between the congruent and incongruent conditions in all experiments, and used such a difference as an index of the priming effect (see Fig 5).

## Experiment 3

In Experiment 1 and 2, we provided evidence that the discrimination of a “cloudy” 3-D structure from motion (SFM) stimulus was more accurate when it was preceded by an unambiguous 3-D SFM with same structure (congruent) than when it was preceded by random dots (neutral) or by a 3-D SFM with different structure (incongruent). However, it is not clear whether the improved accuracy in congruent condition (positive priming) was due to a response bias rather than a perceptual priming effect. To address this problem, here we conducted two additional experiments (Experiment 3A and 3B), in which static objects (3-D drawings (dots) of Cylinder, Sphere, or static noise dots) or semantic words (“cylinder”, “sphere”, and “eeeeeee”) were used as primes to investigate whether the discrimination accuracy of 3-D SFM stimuli can be biased/improved through a top-down modulation. We hypothesized that if the effects observed in Experiment 1 and 2 were mainly due to the object information of the prime SFM stimuli, then similar results would be observed in Experiment 3, as the prime stimuli were either static object shapes, or semantic words.

### Method

In Experiment 3A, static shapes (sphere or cylinder) or static dots (for control condition) were used as the prime stimuli in Experiment 3A. In Experiment 3B, English words (“sphere” or “cylinder”, or “eeeeee” for control condition) were used as the prime stimuli. The ISI in both Experiment 3A and 3B was 2000ms. The duration of target stimuli was the same as in Experiments 1 and 2, and the duration of prime stimuli was 550ms.

### Results and Discussion

#### Experiment 3A

**Fig 6:**
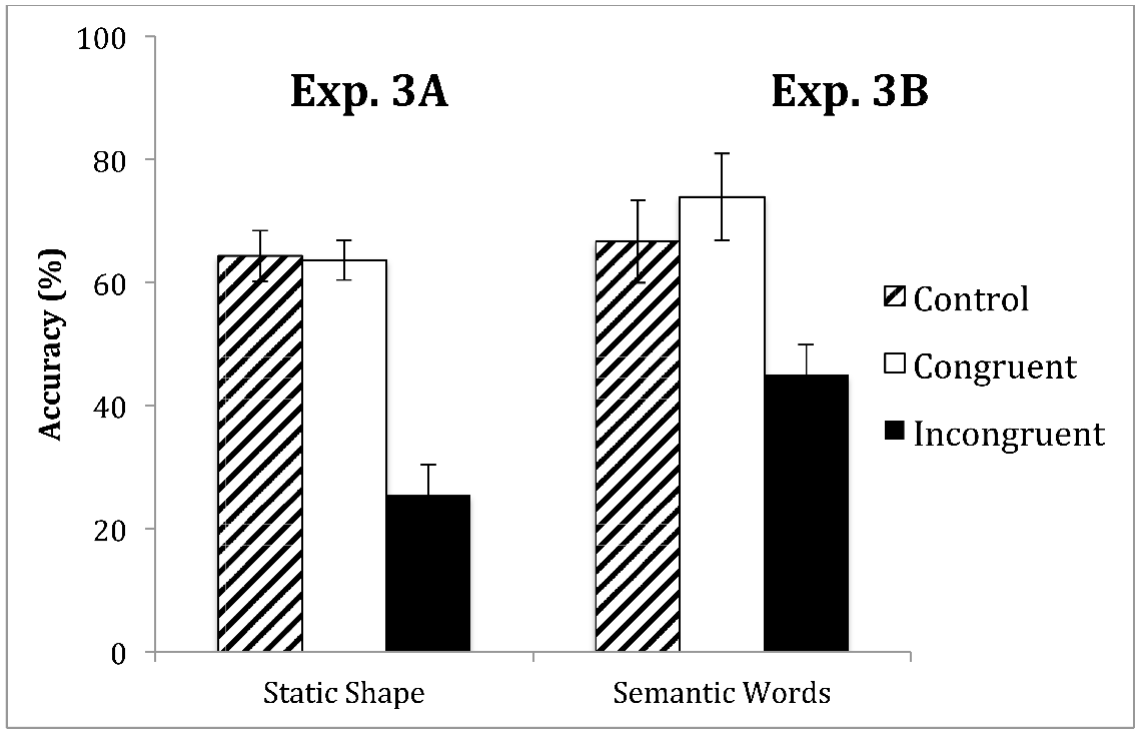
Experiment 3A and 3B. Mean accuracy of the shape judgment about the target SFM stimuli from nine participants in the Experiment 3A and 3B. The data indicated that when preceded by a static shape or a semantic word, there was no priming effect with a prime defining the same shape, but a prime defining the opposite shape had a strong negative impact on the accuracy, suggesting a strong response-bias due to a top-down modulation. Error bars show standard error of mean (SEM).

Overall, participants reported 64.3% (SEM = 4.1%) of target shapes correctly under the control condition. In contrast, under the congruent condition, participants reported 63.7% (SEM = 3.3%) of target shapes correctly, and under the incongruent condition, participants reported 25.5% (SEM = 5.0%) of target shapes correctly (see Fig 6).

Repeated measures ANOVA with the factor of priming conditions (congruent, control, and incongruent) revealed a significant difference (F(2,18) = 35.340, p<0.001). However, in contrast to results of Experiment 1 and 2, post hoc tests revealed that participants performed more accurately under the congruent and control conditions than under the incongruent condition (p<0.00005 and p<0.0001 respectively), but there was no difference between the congruent and control conditions (p>0.89), suggesting the decrease in accuracy under the incongruent condition was due to a conflict from incongruent object information. The lack of difference between congruent and control conditions, and the significant decrease in accuracy under incongruent condition indicated that the static shape cue failed to help to integrate the motion information necessary to improve the accuracy of object recognition of “cloudy” SFM stimuli, but rather affected the recognition of “cloudy” SFM stimuli at a late stage, which might be due to the lack of neural projection from the high level ventral visual cortices (e.g., lateral occipital region, LO) to the dorsal motion-selective regions (e.g., MT/V5).

#### Experiment 3B

In Experiment 3B, we tested how a semantic prime (written English words) would affect the recognition of SFM stimuli. Overall, participants reported 66.6% (SEM = 4.4%) of target shapes correctly under the control condition, and 73.9% (SEM = 4.0%) of target shapes correctly under the congruent condition. In contrast, under the incongruent condition, participants reported merely 45.0% (SEM = 5.7%) of the target shapes correctly.

Repeated measures ANOVA with the factor of priming conditions (congruent, control, and incongruent) revealed a significant difference (F(2,16) = 10.235, p = 0.008). Post hoc tests revealed that participants performed more accurately under the congruent than under the incongruent condition (p<0.003), and more accurately under the control than under the incongruent condition (p<0.04), but there was no significant difference between the congruent and control conditions (n.s.). In line with data from Experiment 3A, the weak difference between congruent and control conditions, and the significant decrease in accuracy under incongruent condition indicated that the preceding semantic cues failed to facilitate the integration of motion information in the dorsal pathway, but rather biased the recognition of a “cloudy” SFM stimulus at a rather late stage (e.g., after LO).

## General Discussion

When a “cloudy” SFM stimulus (due to the presence of a high percentage of noise dots) was preceded by an unambiguous SFM stimulus, a positive perceptual priming effect was found, that is, subjects were more accurate in judging the object shape of the “cloudy” SFM stimulus when it was precede by an unambiguous SFM stimulus that defined the same object shape (congruent), than an unambiguous SFM stimulus that defined a different object shape (incongruent), or a set of random dots (control), while there was no difference between the incongruent and control condition (Figs 2, 3 and 4). In contrast, an opposite effect was found when the “cloudy” SFM stimulus was preceded by a static shape or a semantic word; that is, subjects performed worse when the “cloudy” SFM stimulus was preceded by a static shape or a semantic word of a different object shape (incongruent), than a static shape or a semantic word of the same object shape (congruent), or a neutral cue (control), while there was no difference between the congruent and control condition (Fig 6). Furthermore, the positive priming effect with SFM prime stimuli was still significant six seconds after the offset of the prime stimuli (Fig 5).

In this study, the boundaries of target objects in the target stimuli, as defined by projection and/or dot density, were carefully removed, thus making it impossible for participants to judge the object’s shape without the integration of motion cues. Removing the boundaries also made the shape discrimination task difficult—on average, the accuracy of object perception under the control condition was about 60% in Experiment 1 & 2B, about 70% in Experiment 2A, and between 60–70% in Experiment 3A and 3B. The low performance level in the control conditions may raise concerns that participants might not have perceived the object. One might argue that the priming effect could be due to a response bias; that is, participants failed to perceive the objects in the target stimuli, then tended to respond according to what they saw during the display of prime stimuli. We ruled out this possibility with data from Experiment 3. In Experiment 3, we replaced the SFM primes with semantic or symbolic primes (static images or semantic words). We hypothesized that if the priming effects observed in Exp. 1 and 2 were due to a response bias, then we should observe similar results in Experiment 3. In contrast, the results were opposite to what we observed in Experiment 1 and 2 –there was little to no enhancement in performance with a prime that defined the same shape as the target (congruent condition), but instead a strong negative impact on performance with a prime that defined a shape different from the target (incongruent condition). Such an opposite pattern of modulations suggests that while the more semantic or symbolic primes in Experiment 3 might indeed induce a response bias in or disrupt the recognition of the “cloudy” SFM stimuli at the final decision stage, the SFM primes used in Experiment 1 and 2 likely affected the behavioral performance through a bottom-up perceptual bias at a relatively early perceptual stage, in line with single-unit recording studies [23] that suggest that priming with moving stimuli likely due to modulations at early sensory cortices, probably in the human analogues of motion-selective MT/V5 and FST (the floor of the temporal sulcus area) [24,25].

Recent studies of perceptual priming have suggested that the priming effect is due to modulated neuronal responses at corresponding sensory and other brain regions after an encountering with a previous stimulus [3,26,27]. For instance, motion priming has been attributed to modulated neuronal responses at MT/V5 region [28], and applying repetitive TMS (rTMS) to this region abolishes motion priming [12,29]. Correspondingly, object priming of static image has been linked to neuronal modulations at corresponding regions along the ventral pathway. For example, using a similar prime-target paradigm with a pair of faces [6,30], modulated fMRI responses correlating with the shape similarity between the two faces are revealed in the right fusiform face area (FFA)–an area in the right fusiform gyrus that is central for face processing [31]. In contrast, visual word priming is linked to a change in neural activity in the visual word form area (VWFA)–a region in the left ventral occipitotemporal cortex that is critical for encoding orthographical information of words [32,33]. In contrast to 2D motion that is mainly processed in the dorsal pathway, and static images that are mainly processed in the ventral pathway [34], neuroimaging studies have revealed a wide-spread activations to SFM stimuli, including regions from both the dorsal stream (e.g., MT/V5, FST) and the ventral stream (e.g., lateral occipitotemporal complex, or LOC) [16,35–41]. So what neural mechanisms are responsible for the positive priming under the congruent condition observed in Experiment 1 and 2, and the strong decrease in accuracy under the incongruent condition in Experiment 3?

The SFM stimuli used in the present study can be modeled computationally either based on Euclidean space [30] or affine space [42], which represents an intermediate stage in the processing of SFM stimuli and might be encoded by neurons in the MT/V5, FST (the floor of the temporal sulcus area), parietal regions [16,35–41]. Previous studies have suggested that the motion priming of an ambiguous dynamic stimulus is related to preactivation/stabilization of MT neurons [43]. Here we argue that the perceptual priming effect we observed may be due to the preactivation of either Euclidean or affine structure detectors. Such preactivation would enhance the perception of shape of the target SFM stimulus. Indeed, the lack of priming effect with shapeless motion stimuli in Experiment 1 and the lack of priming with motionless shapes and semantic words under congruent condition in Experiment 3 suggest that, when SFM stimuli were embedded with noise that eliminated the shape information from boundary and dots density, the increase in behavioral performance of recognizing such an SFM stimulus only happened when the same or similar sets of Euclidian or affine detectors were preactivated or stabilized [43], i.e., priming is tightly linked to both motion and structure, in line with previous reports. For instance, it was found that motion priming of SFM stimuli was strongly related to the structure similarity between the prime and target SFM stimuli [18], and shape priming of SFM stimuli was enhanced when the prime and target SFM stimuli have the same motion direction [44]. In a recent study [20] with a unique type of SFM stimuli, 3-D helix, we also found that a static shape or an SFM stimulus with a different rotation axis had little effect on the perception of an ambiguous (bi-stable) SFM stimulus, further supporting a combination of motion and shape is necessary for the observation of SFM priming. This hypothesis of preactivated Euclidian or affine structure detectors is also supported by a recent fMRI-adaptation study of 3-D dynamic object [45]. FMRI-adaptation technique is a technique developed to probe neuronal selectivity [30,46], and the degree of adaptation depending on stimulus similarity, with repetitions of the same stimulus causing the greatest suppression [6,7], thus making fMRI-adaptation an excellent tool of choice to probe neuronal selectivity and invariance in human subjects. Using the fMRI-adaptation paradigm, in addition to adaptation due to motion at the dorsal pathway, Weigelt and colleagues [45] have also found adaptation at ventral pathway (e.g., LOC) when the target stimulus is the same object (as the prime stimulus) of different views along the motion trajectory (but not to the same object of different views outside of the motion trajectory) when the object is rotating in depth, but not when the object is static, suggesting that the neuronal representations of 3-D dynamic objects might be bound to their motion. In other words, the improvement in recognizing a “visual cloudy” SFM stimulus preceded by an unambiguous SFM stimulus of the same object might be due to the preactivations of neurons tuned to the same SFM stimuli (not just shape, but shape plus motion) at both ventral and dorsal pathways, as implicated the lack of increase in accuracy when an SFM stimulus was preceded by a static image that defines the same object shape (Exp. 3A). By contrast, the significant decrease in accuracy under the incongruent condition (and the lack of positive priming under the congruent condition) observed in Exp. 3A&B could be due to a response bias and/or a disruption to the final stage of SFM stimuli processing. These results from Exp. 1–3 thus suggested that perception of a noisy SFM stimulus (i.e., due to the presence of a high percentage of noise dots) can be influenced by varying preceding stimuli at different stages of processing, and future studies are needed to test these hypotheses.
